# Validity and reliability of handgrip dynamometry in older adults: A comparison of two widely used dynamometers

**DOI:** 10.1371/journal.pone.0270132

**Published:** 2022-06-21

**Authors:** Melissa J. Benton, Jefferson M. Spicher, Amy L. Silva-Smith

**Affiliations:** Helen & Arthur E. Johnson Beth-El College of Nursing & Health Sciences, University of Colorado Colorado Springs, Colorado Springs, CO, United States of America; University of Perugia, ITALY

## Abstract

**Background:**

Among older adults, decreased handgrip strength is associated with greater risk of frailty, and loss of physical function, mobility, lean mass, and overall muscular strength and power. Frailty is also associated with sarcopenia, for which handgrip strength measurement has been recommended for diagnostic purposes. Specific cutoff points for diagnosis have been identified, but use of different devices may affect measurement. Therefore to assess validity and reliability, we compared the two most frequently used devices, the Jamar hydraulic and Smedley spring handgrip dynamometers.

**Methods:**

Sixty-seven older (76.2 ± 0.9 years) men (n = 34) and women (n = 33) completed two trials of handgrip strength measurement on sequential days (T1, T2) using both devices in random order. Intraclass correlations were used to assess test-retest reliability, and Bland-Altman analysis was used to assess validity as the level of agreement between devices.

**Results:**

There were significant (*p* < 0.001) relationships between devices at T1 (*r* = 0.94) and T2 (*r* = 0.94) and strong (*p* < 0.001) intraclass correlations were observed for both devices (Jamar = 0.98; Smedley = 0.96), indicating excellent reliability. However, there were significant differences between devices. Strength measured with Jamar was greater than Smedley at both T1 (27.4 ± 1.4 vs. 23.4 ± 1.1 kg, *p* < 0.001) and T2 (25.3 ± 1.4 vs. 21.8 ± 1.2 kg, *p* < 0.001). Bland-Altman analysis confirmed these differences. Subgroup analysis to evaluate the effect of gender and age indicated that in women and old-old (>75 years) participants, differences between devices were closer to zero for both measurements compared to men and young-old (65–75 years) participants.

**Conclusions:**

Our results demonstrate that despite excellent reliability, there is poor agreement between devices, indicating a lack of validity. For use as a diagnostic tool, standardization and device-specific cutoff points for handgrip dynamometry are needed.

## Introduction

In middle and older-aged adults, handgrip strength predicts all-cause and disease-specific mortality, including mortality related to cardiovascular disease, chronic obstructive pulmonary disease, and cancer [1–7]. Furthermore, among older adults in particular, decreased handgrip strength is associated with greater risk of frailty, and loss of physical function, mobility, lean mass, and overall muscular strength and power [8–14]. Handgrip strength is generally recognized as a surrogate measure of whole-body strength and can be used clinically to assess for age-related deterioration in function and health status associated with frailty [3, 8, 14].

Frailty and loss of function and health are also associated with sarcopenia, a geriatric syndrome characterized by loss of muscle and strength [15]. Globally, the prevalence of sarcopenia among adults aged 60 years and over is estimated to be at least 10% [16]. Sarcopenia not only predicts mortality among community-dwelling and acutely ill older adults [17–19], but is also related to functional decline, loss of independence, and hospitalization [20–22]. Exercise interventions can successfully prevent and reverse muscle loss and functional decline [23, 24], but clinical assessment is needed to identify older adults who are at risk [16].

Muscle strength is a biomarker for sarcopenia [25], and handgrip strength measured with dynamometry has been recommended for diagnostic purposes [26]. However, although absolute and precise gender-specific cutoff points for normal handgrip strength have been identified, these cutoff points do not consider potential differences between measurement devices. Currently, there is no universally agreed-upon device or procedure for clinical measurement [26, 27]. In fact, a systematic review of handgrip measurement protocols found incomplete reporting of both the procedures and the devices used [28]. The Jamar hydraulic dynamometer is widely used, but multiple other devices are available for clinical and research purposes [29]. Recent systematic reviews of handgrip strength named at least 10 different devices used for measurement [4, 6, 30]. Among these, the Jamar hydraulic dynamometer and Smedley spring dynamometer were the most frequently identified [4, 6, 30].

There are similarities and differences between the two dynamometers. Both devices weigh approximately 0.66 kg and provide force measurements up to 90 kg. However, the Jamar hydraulic dynamometer displays force using an analog dial with 2-kilogram increments, so smaller more discrete measurements must be interpreted by the operator. By comparison, the Smedley uses a digital display that provides force measurements to the nearest 0.l kg, so operator interpretation is eliminated. Also, both have adjustable handles, to modify grip size, although the Jamar has a concave grip while the Smedley grip is straight. Finally, the Jamar is metal, so the surface temperature can be cooler to touch than the Smedley, which is plastic.

The differences in these two devices may influence the validity and reliability of measurement. To date, we can find only two studies comparing the Jamar and Smedley dynamometers in older adults [31, 32]. Although measurements obtained by the two devices were similar, they were statistically different and influenced by gender [31, 32] and age [31]. Based on gender, differences were greater in women compared to men in one study [31] and greater in men compared to women in the other [32]. Based on age, differences were greater in older compared to younger participants [31]. Moreover, only a single timepoint was used for comparison, so reliability over time could not be evaluated. Therefore, to assess both validity and reliability, we compared sequential grip strength measurements in older adults over a two-day period using a Jamar hydraulic (Patterson Medical, USA) versus a Smedley spring (Takei Scientific Instruments, Japan) handgrip dynamometer. Our secondary aim was to evaluate the effect of gender and age on agreement between devices.

## Materials and methods

### Design

The current study was part of a larger study that has previously been described [33]. Briefly, this was an empirical 2 X 2 design. Participants completed two measurement sessions on consecutive days (T1, T2) using two devices (Jamar, Smedley). T1 measurements were scheduled in the middle of the day when participants were normally fed and hydrated. T2 measurements were scheduled in the early morning on the following day when participants were fasting (i.e., without food or fluids for at least eight hours). This design was specifically intended to elicit loss of muscular strength and function between T1 and T2, which allowed the researchers to assess both reliability and validity when measurements changed over time. The order of testing for each device was randomized between participants and between times. Although interrater reliability for handgrip dynamometry is good to excellent [34], all data were obtained by the same investigator to avoid any potential differences. Ethical approval was obtained from the University of Colorado Colorado Springs Institutional Review Board and all participants provided written informed consent prior to enrollment.

### Participants

Sixty-seven community-dwelling older adults (76.2 ± 0.9 years) volunteered and completed both measurement sessions. Inclusion criteria were age 65 years or older, non-smoking, and able to stand up and ambulate independently or with an assistive device. The only exclusion criterion was the inability to hold the dynamometer and maintain correct positioning during measurement. No participants were excluded from the study. Prior to the first measurement session, all participants completed a demographic and health form.

### Measurements

#### Anthropometrics

Body measurements were obtained using standardized procedures [35]. Before measurement, participants were asked to void and remove their shoes and all excess clothing. Weight was calculated to the nearest 0.2 kg. Height and waist circumference were calculated to the nearest 0.5 cm.

#### Handgrip dynamometry

A brief explanation of the procedure and the two devices, including a demonstration by the researcher, was provided to each participant prior to measurement. For all measurements, the grip width on the Jamar was standardized to the second position (5.0 cm) that has been found to maximize strength production in the majority of adults regardless of age, body mass, or hand dimensions [36, 37]. The grip width of the Smedley was also adjusted to 5.0 cm for uniformity between devices. There is evidence that forearm position affects grip strength [38], so all participants were tested using the same position for both dynamometers. Consistent with recommendations for handgrip dynamometry by the American Society of Hand Therapy [39] and previous research [10, 31], participants sat in a chair with the device held in their dominant hand, their arm supported on a table or other stable surface, their wrist in a neutral position, and their elbow bent at a 90° angle. This procedure has been reported to have high test-retest reliability [40]. Participants then squeezed the device one time, as hard as possible, for 3 seconds. A single attempt was used for each device to avoid muscle fatigue and loss of strength attributed to multiple attempts [37, 41–47], and to avoid pain and discomfort that have been reported with multiple trials [48]. After a 2-minute rest, participants repeated the same measurement procedure with the second device. The maximal force exerted with the Smedley was measured to the nearest 0.1 kg using the digital readout. The maximal force exerted with the Jamar was measured to the nearest 2.0 kg using markers on the analog dial and then estimated by the investigator to the nearest 0.5 kg based on visual inspection of the gauge needle’s position between the 2-kg markers. Both devices were calibrated by the manufacturer prior to the study and maintained according to the manufacturer’s directions throughout the study.

### Statistical analysis

Data were analyzed using SPSS version 27.0 (IBM Corporation, USA) and reported as mean ± SE with 95% CI unless otherwise indicated. Statistical significance was set as *p* < 0.05. Analysis of variance (ANOVA) was used to assess differences at T1 and T2. For a two-group (Jamar and Smedley) comparison using ANOVA for between and within group differences, a sample of 64 participants was determined to be adequate with an alpha < 0.05 (risk of type I error) and beta = 0.8 (risk of type II error) [49]. Pearson correlations were used to evaluate the association between the two devices at T1 and T2, and intraclass correlations (ICC) were used to assess test-retest reliability. For purposes of this analysis, values between 0.8–0.9 were considered good, and values above 0.9 were considered excellent [50, 51]. Bland-Altman analysis was used to assess the level of agreement between devices by plotting differences ± 2 SD against mean values [52]. Plots were visually assessed for characteristics demonstrating good agreement, including mean values close to zero, uniform distribution over the range of measurement, and 95% of differences within ± 2 SD [52]. Finally, to examine the effect of gender and age on agreement, data were stratified by age (young-old 65–75 years, old-old >75 years) and gender (male, female).

## Results

### Participant characteristics

Thirty-four men and 33 women (age range 65–96 years) completed the study. One participant reported use of a walker to ambulate outside of the home and three participants reported use of a cane. All other participants (94%) denied the need for an assistive device for ambulation. Twenty-seven (40%) reported a previous diagnosis of high blood pressure, 17 (25%) reported a diagnosis of heart disease, and 11 (16%) reported a diagnosis of diabetes. There were no differences between men and women for age or body mass index (BMI). However, males had significantly greater (*p* < 0.001) height, body weight, waist circumference and handgrip strength (Table 1).

**Table 1:** Participant characteristics.

	All (N = 67)	Men (n = 34)	Women (n = 33)
Age (years)	76.2 ± 0.9	75.8 ± 1.2	76.7 ± 1.2
	[74.5, 77.9]	[73.4, 78.2]	[74.1, 79.2]
Height (cm)	170.8 ± 1.3	179.3 ± 1.0*	162.0 ± 1.1
	[168.2, 173.4]	[177.2, 181.4]	[159.8, 164.2]
Weight (kg)	76.0 ± 1.9	83.9 ± 2.4*	67.9 ± 2.2
	[72.3, 79.8]	[79.1, 88.8]	[63.5, 72.3]
Body mass index (kg/m^2^)	26.0 ± 0.5	26.1 ± 0.7	25.9 ± 0.8
	[25.0, 27.0]	[24.7, 27.4]	[24.3, 27.5]
Waist circumference (cm)	96.5 ± 1.7	102.5 ± 1.9*	90.2 ± 2.3
	[93.1, 99.8]	[98.7, 106.3]	[85.5, 95.0]
Hand grip strength (kg)			
T1 Jamar	27.4 ± 1.4‡§	34.1 ± 2.0*	20.6 ± 1.1
	[24.7, 30.2]	[30.1, 38.0]	[18.4, 22.8]
T1 Smedley	23.4 ± 1.1‡	29.1 ± 1.5*	17.4 ± 0.9
	[21.1, 25.6]	[26.1, 32.2]	[15.5, 19.3]
Hand grip strength (kg)			
T2 Jamar	25.3 ± 1.4§	31.5 ± 2.1*	18.8 ± 1.0
	[22.5, 28.1]	[27.4, 35.7]	[16.7, 20.9]
T2 Smedley	21.8 ± 1.2	27.4 ± 1.6*	16.1 ± 1.0
	[19.5, 24.1]	[24.1, 30.6]	[14.1, 18.0]

Data presented as mean ± SE [95% CI]

*Significant difference between Men and Women (*p* < 0.001)

**‡**Significant difference between T1 and T2 (*p* < 0.001)

**§**Significant difference between Jamar and Smedley (*p* < 0.001)

### Reliability

**Fig 1:**
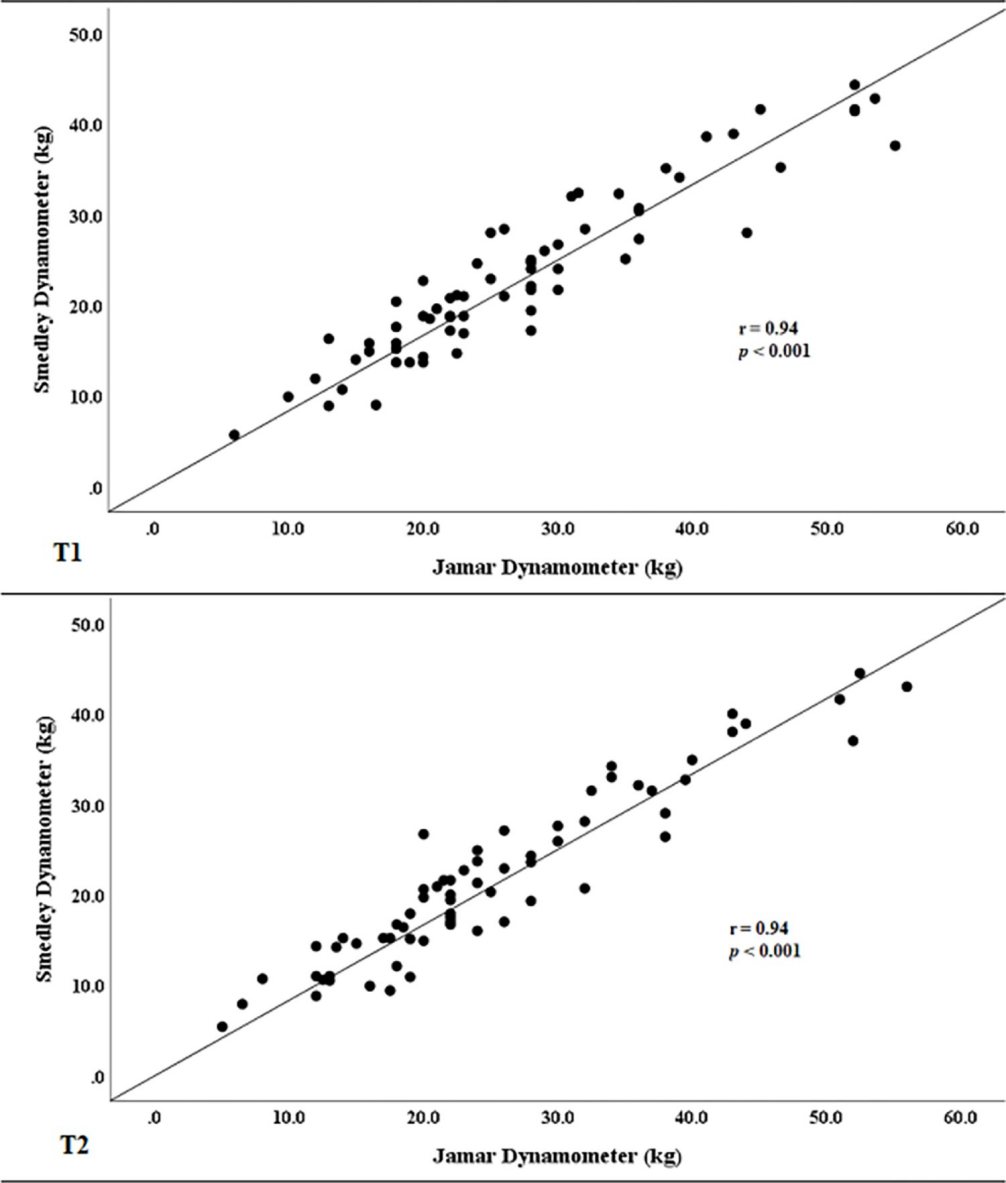
Correlation between devices at T1 (top) and T2 (bottom). Hand grip measurements were strongly and positively correlated at both timepoints.

There were significant within-group differences between T1 and T2 for both devices (Table 1). For the Jamar, strength decreased 8% (*p* < 0.001) and for the Smedley, strength decreased 7% (*p* < 0.001). Despite these differences, both devices were strongly and positively correlated (T1: *r* = 0.94, *p* < 0.001; T2: *r* = 0.94, *p* < 0.001) (Fig 1) and test-retest reliability was excellent (ICC: Jamar = 0.98, *p* < 0.001; Smedley = 0.92, *p* < 0.001).

### Validity

**Fig 2:**
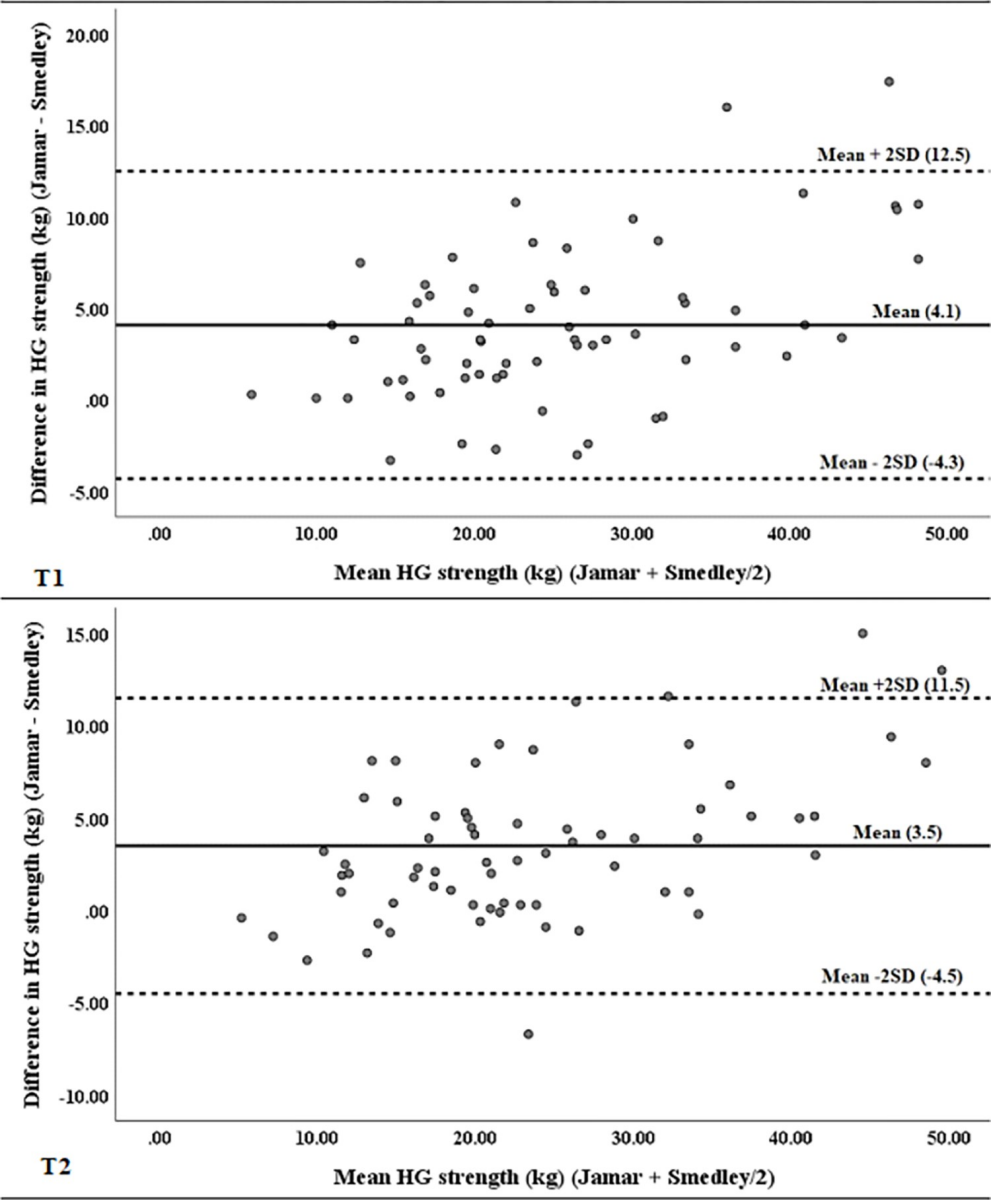
Bland-Altman plots for differences against mean values of hand grip strength measured with the Jamar and Smedley dynamometers at T1 (top) and T2 (bottom).

There were significant between-group differences between devices at both T1 and T2 (Table 1). At T1, there was an average (± SD) difference of 4.1 ± 4.2 kg (*p* < 0.001) between the Jamar and Smedley dynamometers, and at T2 the average (± SD) difference was 3.5 ± 4.0 kg (*p* < 0.001) (Table 2). Bland-Altman analysis indicated poor agreement between the Jamar and Smedley dynamometers at T1 and T2 (Fig 2). Mean values were not close to zero, although they were closer at T2 than at T1, and distribution was not uniform over the range of either measurement. Also, although 97% of differences fell within ± 2 SD at T1, only 94% of differences fell within ± 2 SD at T2.

**Table 2:** Differences in grip strength between the Jamar and Smedley dynamometers at T1 and T2. When stratified by gender and age, differences in measurement were similar between Men and Women, and Young-Old (65–75 years) and Old-Old (>75 years), except at T2 when the difference between devices was significantly diminished for Old-Old compared to Young-Old.

	Difference at T1	Difference at T2
	Mean ± SD	Mean ± SD
	[95% CI]	[95% CI]
All participants (N = 67)	4.1 ± 4.2	3.5 ± 4.0
	[3.1, 5.1]	[2.5, 4.4]
Men (n = 34)	4.9 ± 4.9	4.2 ± 4.5
	[3.3, 6.7]	[2.6, 5.8]
Women (n = 33)	3.2 ± 3.2	2.7 ± 3.2
	[2.0, 4.3]	[1.6, 3.9]
Young-Old (n = 33)	4.9 ± 4.3	4.7 ± 4.4*
	[3.4, 6.4]	[3.2, 6.3]
Old-Old (n = 34)	3.3 ± 4.0	2.2 ± 3.2*
	[1.9, 4.7]	[1.1, 3.3]

*Significant difference between Young-Old and Old-Old (*p* = 0.009)

### Effect of gender and age

**Fig 3:**
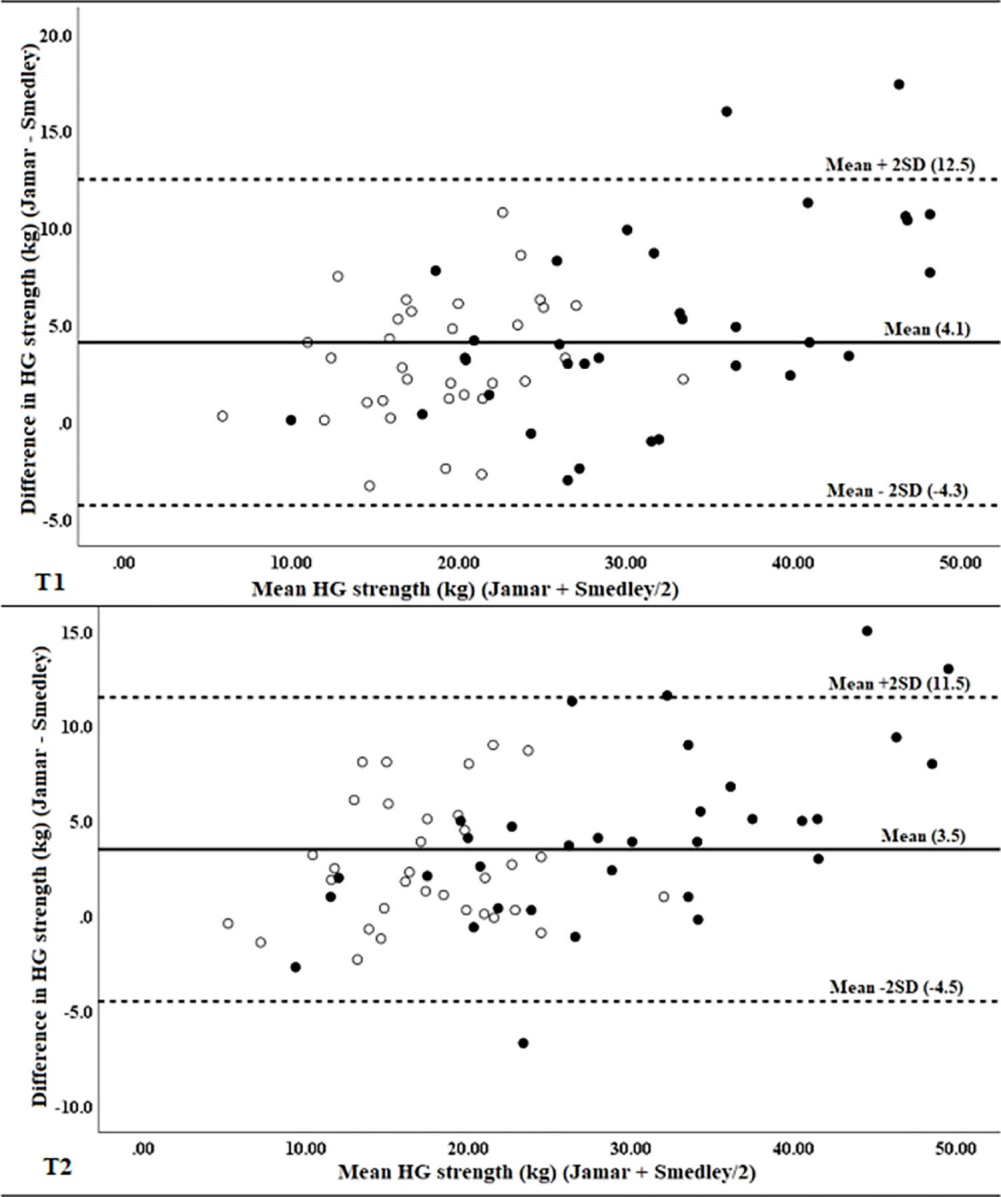
Bland-Altman plots for differences against mean values of hand grip strength measured with the Jamar and Smedley dynamometers at T1 (top) and T2 (bottom) stratified by gender. (women = empty circles, men = solid circles).

When data were stratified by gender, mean differences were not statistically different between men and women at either T1 or T2, indicating no effect for gender, although differences in women were closer to zero for both measurements (Table 2). Visual inspection of Bland-Altman plots found women to cluster at the lower end of the range of strength measurement, reflecting lower absolute grip strength, but with generally similar differences between devices as those observed for men (Fig 3). Interestingly, all differences falling outside of ± 2 SD from mean values were for men.

**Fig 4:**
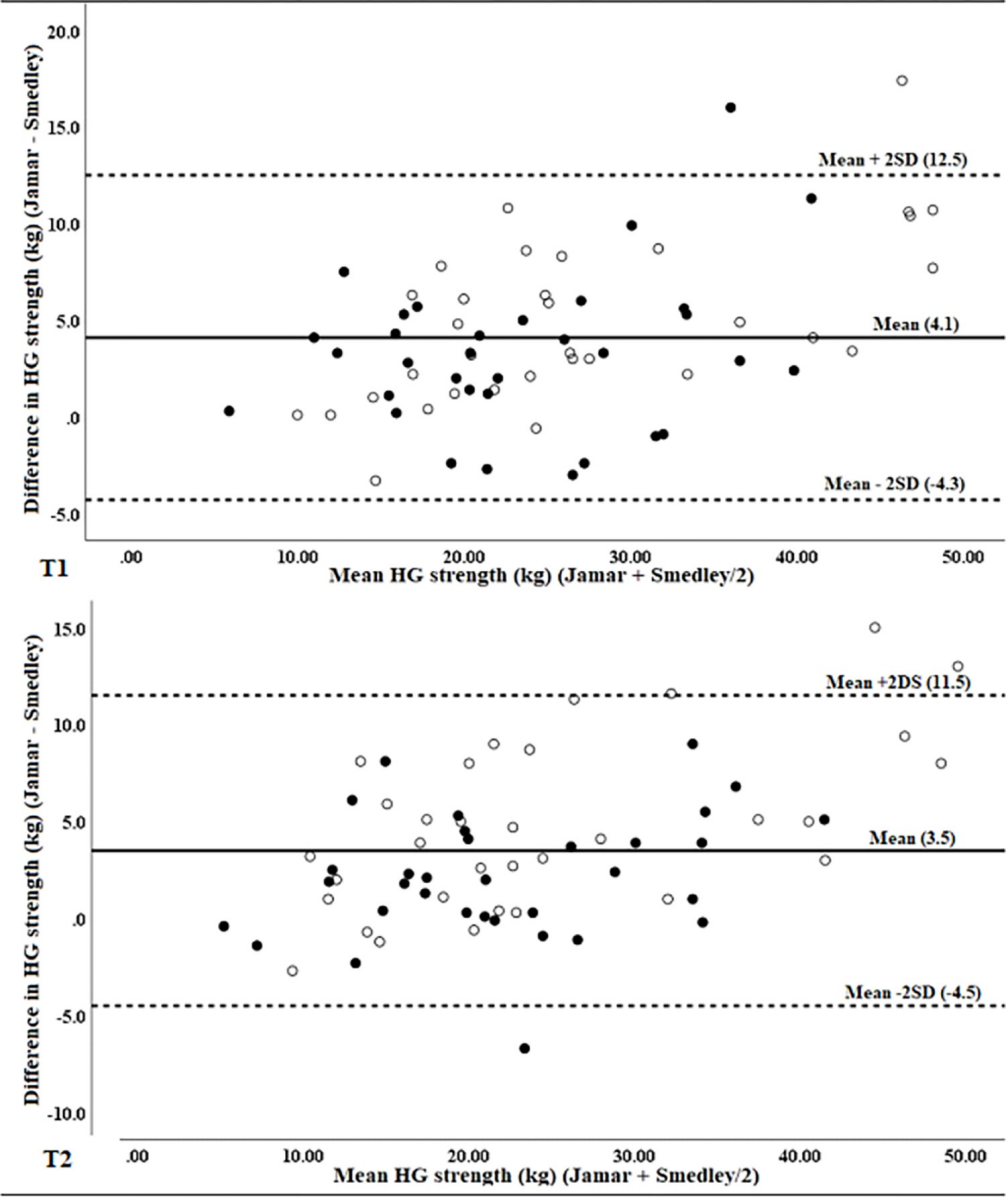
Bland-Altman plots for differences against mean values of hand grip strength measured with the Jamar and Smedley dynamometers at T1 (top) and T2 (bottom) stratified by age. (young-old = empty circles, old-old = solid circles).

When data were stratified by age, mean differences were not statistically different at T1, indicating no effect of age on initial measurement. However, at T2 the mean difference between devices in young-old participants was statistically greater than the mean difference in old-old participants (Table 2), indicating a possible age effect. Visual inspection of Bland-Altman plots found differences in old-old participants to cluster somewhat closer to zero along the range, reflecting smaller mean differences (Fig 4). Clustering was more evident at T2, consistent with the statistically significant difference from young-old participants for that measurement.

## Discussion

To our knowledge, this is the first study to evaluate both the validity and reliability of the Jamar and Smedley handgrip dynamometers that are the most widely used dynamometers for research purposes. Significant differences in handgrip strength were observed between devices at both timepoints, indicating poor validity that was confirmed with visual inspection of Bland Altman plots. However, intra-class correlations for both devices were excellent, indicating good reliability for both devices.

Previously, two studies by Guerra and Amaral [31] and Kim and Shinkai [32] compared the Jamar and Smedley dynamometers at one time point in older adults ranging in age from 65 to 99 years. Guerra and Amaral [31] reported a correlation coefficient of *r* = 0.83 with a mean difference of 3.2 kg, which is similar to but slightly smaller than our current findings. In contrast to our findings, they reported that the level of agreement between the two devices was poorer for women compared to men, and old-old compared to young-old. In our sample, women demonstrated better agreement than men, and old-old participants demonstrated better agreement than the young-old. Participant differences may somehow have influenced discrepancies in our findings. Our sample was somewhat younger, with an average age of 76.2 years, compared to 79.2 years for their sample. Furthermore, our sample was evenly distributed between men and women, while theirs was predominantly (76%) female.

By comparison, Kim and Shinkai [32] reported a correlation coefficient of 0.98, which was similar to but somewhat larger than our findings. They also observed a larger mean difference in men (3.09 kg) compared to women (2.6 kg) that was statistically significant. Our results were similar, in that differences were smaller in women compared to men, although they did not achieve statistical significance. The difference in significance may again be related to the gender composition of the study samples. Sixty percent of the sample tested by Kim and Shinkai [32] were males and only 40% were females. When the results of all three studies are compared, the sample evaluated by Guerra and Amaral [31] that was majority female exhibited significantly greater differences in agreement among women; our sample that was evenly balanced by gender exhibited non-significant differences in agreement between genders; and the sample evaluated by Kim and Shinkai [32] that was predominantly male exhibited significantly greater differences in agreement among men.

Guerra and Amaral [31] attributed discrepancies in measurement to an interaction between participants and dynamometer characteristics. As previously described, there are both similarities and differences between the Jamar and Smedley dynamometers. Although they may be considered subtle, these differences could be sufficient to cause the discrepancies in measurement observed in all three studies, while differences in our samples may have been sufficient to cause the inconsistent effects of gender and age that were observed. For example, the ability to exert handgrip strength is influenced by pain or discomfort [13], so the design of each dynamometer may be of importance. We believe that more research in this area is needed.

It has been suggested that increasing handgrip strength may independently improve physical and functional resilience, resulting in improved health outcomes [2]. A meta-analysis of 25 studies, including almost 200,000 adults with an average age of 65 years or older, demonstrates that increasing handgrip strength by only 1 kg reduces mortality due to heart disease by 30% [6]. This is an intriguing finding, but without clear guidelines for handgrip strength measurement, accurate clinical assessment is difficult and appropriate implementation of interventions is problematic.

Current diagnostic criteria for sarcopenia in older adults differentiate handgrip strength cutoff points based on gender [26, 27]. This is supported by the absolute differences between men and women we observed. However, the diagnostic cutoff points ignore differences between devices that are highlighted by our current findings. Specific cutoff points of <27 kg for men and <16 kg for women are recommended by the European Working Group on Sarcopenia in Older People (EWGSOP) [26], and <28 kg for men and <18 kg for women are recommended by the Asian Working Group for Sarcopenia (AWGS) [27], but they are not specific to the device used for measurement. By comparison to handgrip strength, the AWGS has now differentiated cutoff points for muscle mass that are device specific [27]. They differentiate cutoff values for skeletal muscle based on either dual-energy x-ray absorptiometry (DXA) or bioelectrical impedance analysis (BIA), in recognition that both are widely used technologies with numerous advantages but without absolute agreement. Based on our findings, we believe that device-specific cutoff points for handgrip strength are also needed. This is consistent with the EWGSOP’s key recommendation that simple, specific cutoff points for measures used to identify sarcopenia be developed [26].

We recognize that there are limitations to our study. Our sample size was relatively small, although it exceeded the sample size needed for adequate power. Furthermore, a small sample is primarily of concern due to the risk of a type II error. The significant differences we observed appear to rule out this concern in relation to our findings. It is also possible that use of another procedure for handgrip measurement may have obviated differences between the two devices. As previously noted, there are multiple procedures reported in the literature that include variations in arm positioning and the number of attempts used for measurement. Since our objective was solely to compare devices, we chose to to minimize the potential effect of position on measurement by testing all participants in the same position. It is possible that use of the seated position to test the Smedley device may have influenced our results, but we do not believe it did. Our decision was based on concerns for safety since participants were older and one of our measurements was taken early in the morning when we anticipated they would be weaker and less steady. For that reason we chose to have them test in a seated position for safety. This is not inconsistent with the AWGS 2019 Consensus Update [27] that recommends (but does not require) a standing position for measurement with the Smedley, based specifically on the US National Health and Nutrition Examination Survey (NHANES) protocol. However, AWGS notes [27] (page 303) that “*The NHANES protocol also permits sitting for people who are unable to stand unassisted*.” AWGS is also very specific that “*sitting is preferable if older people cannot stand unassisted*.” The participants in our study were old (average age 76 years) and on the second day, measurements were taken early in the morning while fasting in order to elicit loss of strength, which we believed would provide a stronger and more novel assessment of reliability over time (test-retest reliability). Hence, we made an *a priori* decision to have participants sit during measurements to promote comfort and avoid any risk of imbalance that could have resulted in a fall. Also, although the standing position with arm extended is used with the Smedley device, that position is not universal. In a systematic review of handgrip strength protocols by Sousa-Santos & Amaral [28] nine studies reported use of the Smedley (Takei) digital dynamometer. Of these, six (66%) used a standing position, two (22%) used a sitting position, and one (11%) did not report the position used. Furthermore, Guerra and Amaral [31] used the same position for testing that we employed, while Kim and Shinkai [32] tested the Jamar in a seated position and the Smedley standing, yet our results are consistent, which appears to negate the effect of positioning on our study outcomes. The number of attempts used may also have influenced our findings. Because multiple attempts have been found to be similar to a single attempt [37, 41–48, 53], we chose to minimize participant burden by using only a single attempt for each device. By comparison, the two previous studies used two [32] and three [31] trials, yet our findings regarding agreement between devices were consistent, which again appears to negate the effect of single versus multiple attempts on outcomes. Finally, there is precedent in previous research. Bohannon and Schaubert [54] used a single attempt with a Jamar dynamometer to evaluate test-retest reliability of grip-strength measurement in older adults, and more recently Yoshida and colleagues [55] used a single attempt with a Smedley to evaluate grip strength for diagnosis of sarcopenia. However, the influence of these variations should be further explored in future research.

## Conclusion

The mean differences we observed between the Jamar and Smedley, two of the most widely used handgrip dynamometers, could result in misdiagnosis either for or against sarcopenia (i.e., either a false positive or a false negative). Nevertheless, as a diagnostic tool, handgrip dynamometry has numerous advantages including low cost, portability, rapid results, and easy use. However, without device-specific cut points or a universally agreed-upon device for handgrip dynamometry, sarcopenia treatment and research may be impeded. Individual labs and clinics may not have universal access to all types of devices available, so device-specific cutoff points for handgrip dynamometry would appear to have the greater advantage and are recommended.

## Supporting information

S1 Data (PDF)
