# Supplementary material for: Validity and reliability of handgrip dynamometry in older adults: A comparison of two widely used dynamometers
Source: PLoS One. 2022 Jun 21;17(6):e0270132. doi: 10.1371/journal.pone.0270132 (PMC9212147; doi:10.1371/journal.pone.0270132)
Supplement: S1 Data — (PDF) [file pone.0270132.s001.pdf]

Age	Gender	Height	Wgt#1	BMI1	WC	JamarHG1	JamarHG2	SmedHG1	SmedHG2
79	1	166	90	32.7	114	16	12	14.9	14.3
78	1	173	54.4	18.2	68	18	17.5	13.7	15.2
75	1	158	57.6	23.1	81.25	28	26	19.4	17
73	1	156	67.2	27.6	100	34.5	32.5	32.3	31.5
70	1	170	61	21.1	66	28	26	24.7	22.9
74	1	163	74.8	28.1	101	20	20	18.8	14.9
76	1	163	87	32.8	95.75	30	24	24	24.9
79	1	165	53.2	19.5	72.5	20	19	14.3	10.9
75	2	188	83.6	23.6	97	52	51	41.4	41.6
72	2	185	62.2	18.1	81	22.5	20	21.1	20.6
67	2	180	92.4	28.5	102.5	52	52.5	41.6	44.5
92	2	170	61.4	21.3	93	30	28	26.7	24.3
71	2	183	71.8	21.5	90	52	52	44.3	37
86	1	161	67.6	26.1	94.5	20.5	17	18.5	15.2
70	1	163	85	32	102.5	15	14	14	15.2
79	2	178	74.6	23.5	100	44	38	28	29
71	1	172	75	25.3	95	22	24	17.2	16
71	2	185	106.6	31.2	115	39	38	34.1	26.4
77	1	150	61.8	27.5	89	14	13	10.7	10.5
96	1	157	61.4	24.9	94.5	16	15	15.8	14.6
65	2	182	79.6	24	96	45	43	41.6	40
87	1	162	55	21	79	23	22	21	16.7
81	2	185	93.6	27	105.5	38	34	35.1	34.2
76	1	170	69.6	24.1	81.5	22	20	20.8	19.7
77	1	167	71.2	25.6	93.5	6	5	5.7	5.4
89	1	159	74.4	29.4	99	20	21.5	22.7	21.6
78	1	158	56.6	22.7	74	19	16	13.7	9.9
84	1	152	68.6	29.7	95	13	12.5	8.9	10.6
72	1	169	80.2	28.1	98	28	19	22.1	17.9
85	1	152	59.2	25.7	91.5	16.5	6.5	9	7.9
74	1	168	69.6	24.6	89	25	23	22.9	22.7
65	1	165	60.6	22.3	87	28	28	17.2	19.3
78	1	154	71	29.9	98.75	18	22	20.4	20
78	1	164	61.2	22.8	82	21	21	19.6	20.9
73	1	163	107.8	40.6	133.5	12	12	11.9	8.8
77	2	183	99	29.6	119	35	32	25.1	28.1
74	1	160	68.8	26.9	96.5	13	13.5	16.3	14.2
69	1	164	70.2	26.1	94.5	23	17.5	16.9	9.4
73	2	175	75.8	24.8	97	29	25	26	20.3
76	1	172	77	26	93	26	22	21	17.5
88	1	156	56.8	23.4	84	18	18	15.2	16.7
65	1	160	56.4	22.1	81	28	24	21.7	21.3
85	2	170	68.8	23.8	94.5	36	36	30.7	32.1
74	2	190	84.4	23.4	98	43	40	38.9	34.9
79	2	177	78.3	25	103	31	30	32	27.6
70	2	185	85.4	25	99	22.5	30	14.7	25.9

70	2	185	95	27.8	110	55	56	37.6	43
80	2	170	77.6	26.9	108	36	39.5	30.4	32.7
78	2	184	87.3	25.8	108	46.5	44	35.2	38.9
72	2	180	71.5	22.1	90	24	22	24.6	19.4
79	2	183	97.7	29.2	111	28	20	24	26.7
67	2	176	95.9	31	98	30	32	21.7	20.7
76	2	171	89.2	30.5	108	23	24	18.8	23.7
74	2	179	70.9	22.1	96	28	22	25	21.6
73	2	174	73	24.1	96	36	22	27.3	17
68	2	185	110.4	32.3	126	10	8	9.9	10.7
69	2	180	89.3	27.6	97	18	12	17.6	11
74	2	178	83	26.2	103	22	13	18.8	11
92	2	162	64.3	24.5	90	41	37	38.6	31.5
67	2	179	117.7	36.7	133	53.5	43	42.8	38
69	1	161	60.8	23.4	81	20	18	13.7	12.1
82	2	179	88.8	27.7	114	26	26	28.4	27.1
88	2	178	67.7	21.4	91.5	22	18.5	18.7	16.4
81	2	183	93.1	27.8	110	31.5	34	32.4	33
78	2	179	94.4	29.4	112	25	22	28	17.9
77	2	174	68.8	22.7	93	32	28	28.4	23.6
69	1	154	49.3	20.8	72	18	19	15.8	15.1
